# Analysis of psychometric properties of the modified SETQ tool in undergraduate medical education

**DOI:** 10.1186/s12909-017-0893-4

**Published:** 2017-03-16

**Authors:** Ahmed Al Ansari, Kathryn Strachan, Sumaya Hashim, Sameer Otoom

**Affiliations:** 1 Bahrain Defense Force Hospital, Off Waly Alahed Avenue, P. O. Box-28743, Riffa, Kingdom of Bahrain; 2 RCSI Bahrain, Arabian Gulf University, P.O. Box 15503, Adliya, Kingdom of Bahrain; 3 General Surgery Arabian Gulf University (AGU), Manama, Bahrain

**Keywords:** SETQ, Bahrain, Validity, Reliability

## Abstract

**Background:**

Effective clinical teaching is crucially important for the future of patient care. Robust clinical training therefore is essential to produce physicians capable of delivering high quality health care. Tools used to evaluate medical faculty teaching qualities should be reliable and valid. This study investigates the psychometric properties of modification of the System for Evaluation of Teaching Qualities (SETQ) instrument in the clinical years of undergraduate medical education.

**Methods:**

This cross-sectional multicenter study was conducted in four teaching hospitals in the Kingdom of Bahrain. Two-hundred ninety-eight medical students were invited to evaluate 105 clinical teachers using the SETQ instrument between January 2015 and March 2015. Questionnaire feasibility was analyzed using average time required to complete the form and the number of raters required to produce reliable results. Instrument reliability (stability) was assessed by calculating the Cronbach’s alpha coefficient for the total scale and for each sub-scale (factor). To provide evidence of construct validity, an exploratory factor analysis was conducted to identify which items on the survey belonged together, which were then grouped as factors.

**Results:**

One-hundred twenty-five medical students completed 1161 evaluations of 105 clinical teachers. The response rates were 42% for student evaluations and 57% for clinical teacher self-evaluations. The factor analysis showed that the questionnaire was composed of six factors, explaining 76.7% of the total variance. Cronbach’s alpha was 0.94 or higher for the six factors in the student survey; for the clinical teacher survey, Cronbach’s alpha was 0.88. In both instruments, the item-total correlation was above 0.40 for all items within their respective scales.

**Conclusion:**

Our modified SETQ questionnaire was found to be both reliable and valid, and was implemented successfully across various departments and specialties in different hospitals in the Kingdom of Bahrain.

**Electronic supplementary material:**

The online version of this article (doi:10.1186/s12909-017-0893-4) contains supplementary material, which is available to authorized users.

## Background

A robust clinical training experience is essential in producing physicians capable of delivering high quality health care. Effective clinical teachers have been described in previous studies as clinically knowledgeable, compassionate, having strong integrity and possessing solid teaching skills. Additionally, effective teachers actively involve their students in patient care and provide constructive feedback and guidance [1].

Review studies have found that over 32 different instruments have been developed to assess clinical teachers [2]. A small number of these instruments assesses only student performance [3]. Some of those instruments are not validated, while some studies argue that instruments must be validated against the specific context they are being applied to [4].

Such questionnaires are essential for the continuous development of medical students’ education and for the ongoing improvement of clinical teaching skills. Because students at different stages of their educational careers may be looking for different attributes in a clinical teacher [5, 6], these instruments should be administered to a wide variety of samples at different points in the learning process. This will support the criterion validity of the instrument. It has been suggested that for an instrument to be used by specific groups or in different cultural and educational contexts, the instrument should be continuously revalidated and updated. Recent psychometric studies underscore the importance of viewing validation as an ongoing process [2, 6].

One of these published instruments is the “System for Evaluation of Teaching Qualities” (SETQ) [7]. We chose this instrument because its domains covered most of the criteria we had identified beforehand in a table of requirements for our purpose of evaluation. In addition, the small number evaluations needed to produce reliable results were considered a strength of the SETQ instrument. As a well-established, reliable and valid instrument we felt that the SETQ provide a good basis for further modification in order to fit the evaluation by medical students [7, 8].

The original SETQ instrument has been used extensively among resident doctors in the Netherlands across different hospitals and different departments, such as anesthesiology, obstetrics and gynecology but was not applied in the Middle Eastern settings or with undergraduate students [7]. However, we are applying it in this Middle Eastern setting to test its ability to produce viable results in a different setting.

Three phases are involved in the modified SETQ system: (i) data collection and evaluation, (ii) individual feedback reports generated for each faculty and (iii) discussing the individualized reports with each individual faculty. During the first phase, responses are collected from the students who evaluate the clinical teachers, and a self-evaluation form is collected from the clinical teachers themselves. The second phase consists of data analysis and generation of individualized reports for each of the clinical teachers. The last phase involves discussing the reports with each individual faculty, Chief of Medical Staff in each hospital and with the Department Head. In the future, a fourth phase may be added to re-evaluate the clinical teachers and compare the differences between their previous performance and their performance after feedback [8].

The original SETQ instrument was developed based on the Stanford Faculty Development Program SFDP26 and consisted of 23 items and covered the following 5 domains: learning climate, professional attitude towards and support of residents, communication of goals, evaluation of residents, and giving feedback. However, the last domain in our modified SETQ instrument focuses on promoting self-directed learning which was obtained from the original SFDP26 instrument. The original SFDP26 instrument was developed in the USA and covers the following seven categories: establishing the learning climate, controlling a teaching session, communication of goals, encouraging understanding and retention, evaluation, feedback, and self-directed learning [9].

The aim of this study was to investigate the psychometric properties of modification of the SETQ instrument in the clinical years of undergraduate medical education.

## Methods

### The modified SETQ instrument

In our study, we have developed the modified SETQ instrument utilizing the SFDP26 and the original SETQ instruments. The SFDP26 and The System for Evaluation of Teaching Qualities (SETQ) are two instruments that have been validated and widely accepted by the academic community. The SFDP26 is one of the instruments used extensively in the USA for teachers of Undergraduate Medical Education. The original SFDP26 consisted of 26 items and covered the following seven domains: establishing the learning climate, controlling a teaching session, communication of goals, encouraging understanding and retention, evaluation, feedback, and self-directed learning [9]. The SETQ instrument, was used in The Netherlands, primarily for residency training at the Post Graduate Medical Education level. It was originally developed based on the SFDP26 instrument and consisted of 23 items covering the following 5 domains: learning climate, professional attitude towards and support of residents, communication of goals, evaluation of residents, and giving feedback.

However, the first five domains in our modified mSETQ instrument shared the same domains of the original SETQ. While maintaining the main domains of the SETQ instrument, we added an additional domain named “promoting self-directed learning” as we felt this domain should be given weightage at par with other domains of the instrument at the undergraduate medical student level. This domain was derived from the SFDP26 instrument. The subscales of the modified SETQ version were derived from both, the original SETQ and the SFDP26 instrument which were compatible with our undergraduate medical education.

Few modifications have been done. For instance, the first domain title was changed from learning climate to teaching and learning environment. The total number of items in the first domain is 6 in our instrument, whereas in the SETQ is five. Moreover, items 1, 2 and 4 in the ‘Teaching and Learning Environment’ in our instrument were similar to items 1,2 and 5 in SETQ. On the other hand, items 3, 5 and 6 in our instrument (keeps to teaching goals; teaches on ward rounds, at clinics, and operating room; covering all the topics which are in the curriculum) were constructed items based on our table of specification and the expert opinion. The same methodology was applied in constructing the remaining domains.

After developing the modified version to suit medical students, face and content validity were established by expert opinion and with the use of a table of specification. We addressed face and content validity by sending the instrument to six experts in the field to review both the content and format of the modified instrument and to judge whether or not it was appropriate to assess medical students. In addition to the expert opinion, the questions in the survey were assessed against the table of specification that was constructed by the authors.

The survey comprised 25 items and assessed six major domains rated on a 5-point Likert scale. The items on the instrument had a 5-point response scale in the form of: “1 = strongly disagree; 2 = disagree; 3 = neither agree nor disagree; 4 = agree; 5 = strongly agree,” with an option of “unable to assess” (UA).

For each question of the survey, the percentage of individuals who responded “unable to assess,” was calculated to identify the viability of the items and the score profiles. Items in which more than 20% of responders selected “unable to assess” were considered in need of revision or deletion.

### Piloting the study

In November 2014, a pilot study was conducted to evaluate students’ ratings of their clinical teachers via a paper-based questionnaire and to examine the content validity of the modified SETQ instrument. We distributed 88 surveys and received 70 completed responses. This pilot study revealed many incomplete questionnaires with missing information and some feedback regarding the content of questions used in the instrument. To correct this issue, a second pilot was conducted in March 2015 after modifying some of the questions and an electronic questionnaire was used with mandatory responses for each item. However, a very low response rate was achieved, with only three surveys being returned over a 2-week period.

Following these two pilot studies, we concluded that the electronic-based questionnaire might not be feasible in our setting. We decided to change the strategy by using printed paper-based packets that included important details such as rotation date, clinical teacher, hospital name, and student information. Students were given the option of providing their details or omitting them from their responses, which were rendered confidential with identifiers removed before reaching the researcher. In addition to the questionnaires, each packet included an information sheet, detailing the research purpose and a mandatory consent form (Additional file 1).

### Study population and settings

We invited 298 medical students from the clinical years and 102 clinical teachers working in four teaching hospitals in the Kingdom of Bahrain to participate in the modified SETQ study. The clinical teachers were working in different departments, including surgery, medicine, psychiatry, paediatrics, obstetrics and gynaecology, and pathology. 88 students received packets via their clinical coordinators during their rotations, while 95 packets were distributed to students during campus lectures. Students in their clinical rotations were asked to return their completed surveys to their clinical coordinators, while students on campus were encouraged to participate in the study via email from the Director of Senior Cycle. A combined total of 1094 surveys were completed and were transferred into an electronic format by the research administrator.

Because conversion to an electronic format was time consuming and labour intensive, an electronic version of the survey was administered to the remaining 115 students, with many reminders and follow-ups. Of these electronic surveys, 67 were completed, for a combined total of 1161 questionnaires evaluating 105 clinical teachers were based on rotation experiences at four different hospitals. Data collection lasted 6 months from November 2014 through April 2015.

### Analytical strategies

Data was analyzed using SPSS version 20.0. For the pilot study, each research question underwent a number of statistical analyses. The feasibility of the questionnaire was analyzed using the response rate, the average time required to complete the form, and the number of raters required to produce reliable results. For each survey question, the percentage, mean, and standard deviation of UA responses were calculated to identify the viability of items and score profiles. Items with > 20% UA responses were deemed in need of revision or deletion following past findings [10].

Descriptive statistics were generated for clinical teachers and students. To assess the validity of the modified (SETQ) instrument, an exploratory factor analysis was conducted to identify which items on each survey belonged together, becoming a factor or scale. In our study, items were intercorrelated using Pearson product moment correlations. The correlation matrix was then decomposed into principal components, which were rotated to the normalized varimax criterion. The primary loading for each item determined which factor the item would belong to. The number of factors extracted was based on an a priori specification of six factors [11].

After extracting the factors, key domains were identified for improvement in each factor through feedback, and the items in each factor provided specific information about particular behaviors (e.g., whether the clinical teacher offers suggestions for improvement). This analysis made it possible to determine whether the instrument items were aligned with the appropriate constructs (factors) as intended. Each item was assigned to the factor in which it loaded with a loading factor of at least 0.40. If an item loaded in more than one factor (cross-loading), the item was assigned to the highest-loaded factor [12].

Instrument reliability (stability) was assessed. The internal consistency reliability coefficient was examined by calculating Cronbach’s alpha for the total scales and for each factor. This calculation provided an assessment of the overall internal consistency for each instrument and for each factor within the instrument [13]. A Cronbach’s alpha of 0.70 was considered acceptable.

To examine the homogeneity of each composite scale, we calculated item-total correlations corrected for overlap [14]. We considered an item-total correlation coefficient of < 0.3 as evidence that the item was not measuring the same construct measured by the other composite scale items. In addition, Pearson’s correlation coefficients were used to estimate the inter-scale correlations that determine the degree of overlap between the scales [15].

Finally, we used previously reported data to calculate the number of students required to evaluate each clinical teacher to produce a reliable assessment [15].

## Results

A total of 125 medical students completed 1161 evaluations of 105 clinical teachers based on their rotation experiences at four different hospitals. Fifty-seven clinical teachers completed self-evaluation form as well. Students completed 11.2 assessments per clinical teacher (Additional file 2).

Characteristics of both students and clinical teachers are presented in Table 1.

**Table 1:** Characteristics of students and clinical teachers who participated in the evaluation

	Students’	Clinical tutors
Number invited	298	125
Number of responses	125	105
Response rate	42%	84%
Total number of evaluation	1161	NA
Total number of self-evaluation	NA	57 (56%)
Percentage respondents who are female	61%	NA
Mean number of each clinical tutor evaluated by students	11.3	1
Number of clinical tutors per hospitals		
Hospital A	NA	38
Hospital B	NA	27
Hospital C	NA	32
Hospital D	NA	8
Number of students per year of clinical rotation		
Intermediate Cycle 3 (IC3)	28	NA
Senior Cycle 1 (SC1)	45	NA
Senior Cycle 2 (SC2)	52	NA
Number of clinical tutors per department		
Medicine	NA	38
Surgery	NA	32
Obstetrics & Gynecology	NA	13
Pediatrics	NA	12
Psychiatry	NA	8
Pathology	NA	2
The cut-off point according to the 1st quartile		
Total instrument	3.80	NA
Teaching & Learning environment	3.92	NA
Professional attitude towards Students	3.79	NA
Communication of Goals	3.83	NA
Evaluation of students	3.74	NA
Feedback	3.88	NA
Promoting self-directed learning	3.95	NA
Number of years of experience in teaching		
Less than 1 year	NA	28
2 years of experience	NA	32
3-4 years of experience	NA	24
5-6 years of experience	NA	30
7-8 years of experience	NA	11
Nationality of the clinical tutors		
Bahrainis	NA	60%
Egyptians	NA	8%
Indian	NA	4%
British	NA	4%
Irish	NA	3.2%
Pakistani	NA	3.2%
Algerian, French, Nigerian, South African, Sudanese and Syrian	NA	4.8%

Students assessed their clinical teachers on six domains. The cutoff point was set according to the 1^st^ quartile, and it was 3.8, whereby any results below this were considered at-risk and in need of improvement. The cutoff point according to the 1^st^ quartile was measured for the subscales and it was as follows: 3.92 for teaching and learning environment, 3.79 for professional attitude towards students, 3.83 for communication of goals, 3.74 for evaluation of students, 3.88 for feedback, and 3.95 for promoting self-directed learning. On a hospital-wide level, all the clinical tutors teaching in four of the hospitals scored well above a 3.8, with a range from 4.05 to 4.31. The two highest scoring domains across the clinical tutors were the teaching and learning environment and professionalism toward students, while the lowest scoring domains were communication of goals and evaluation of students.

### Feasibility

The response rates were 42% for the student evaluations, and 57% for the self-evaluation by the clinical teachers. The average time needed to fill out the questionnaire was three minutes and the low number of evaluations needed for reliable assessment (4 raters) indicates the feasibility of the modified SETQ instrument (Additional file 3).

### Reliability and validity of the modified SETQ instrument

Six domains were identified based on the factor loading from the exploratory factor analysis. The whole instrument was found to be suitable for factor analysis [Kaiser-Meyer-Olkin (KMO) = 0.953; Bartlett test significant, *P* < 0.001]. The factor analysis showed that the data on the questionnaire decomposed into six factors that represented 76.7% of the total variance: teaching and learning environment (items 1 to 6), professional attitude towards students (items 7 to 10), communication of goals (items 11 to 13), evaluation of students (items 14 to 18), feedback (items19 to 22), and promoting self-directed learning (items 23 to 25). The factor loadings in the student analysis were all above 0.60, except for four items in the scale, Table 2.

**Table 2:** Characteristics of composite scales and items, with internal consistency reliability coefficient and corrected item-total correlation

		Factor loading on primary scale	Internal consistency reliability cronbach’s α ^a^		Corrected item total correlation ^b^	
Nr	Scale and items	Student’s evaluation	Student’s evaluation	Clinical tutors evaluation	Student’s evaluation	Clinical tutor self-evaluation
Teaching & Learning environment			0.95	0.88		
TL1 Encourages students to participate actively in discussions		0.674			0.847	0.831
TL2 Stimulates students to bring up problems		0.654			0.873	0.676
TL3 Keeps to teaching goals; avoids digressions		0.653			0.850	0.710
TL4 Prepares well for teaching presentations and talks		0.600			0.874	0.829
TL5 Teaches on ward rounds, at clinics, and operating room		0.569			0.845	0.745
TL6 Covering all the topics which are in the curriculum		0.494			0.859	0.554
Professional attitude towards Students			0.94	0.91		
P1 Listens attentively to students		0.713			0.847	0.833
P2 Is respectful towards students		0.790			0.770	0.848
P3 Is available regularly for the students		0.699			0.827	0.689
P4 Is easily approachable for discussions		0.771			0.815	0.825
Communication of Goals			0.97	0.81		
C1 States learning goals clearly		0.697			0.886	0.721
C2 Prioritizes learning goals and topics		0.675			0.886	0.671
C3 Debriefing the learning goals periodically		0.689			0.882	0.594
Communication of Goals			0.96	0.91		
E1 Evaluates student’s specialty knowledge regularly		0.653			0.886	0.879
E2 Evaluates student’s analytical abilities regularly		0.676			0.880	0.699
E3 Evaluates student’s application of knowledge to specific patients		0.657			0.876	0.813
E4 Evaluates student’s medical skills regularly		0.554			0.868	0.758
E5 Evaluates student’s, communication, and professionalism during patient encounter		0.497			0.876	0.734
Feedback			0.96	0.84		
F1 Regularly gives constructive feedbacks to students		0.648			0.891	0.726
F2 Explains why students are incorrect		0.609			0.884	0.655
F3 Offers suggestions for improvement		0.663			0.890	0.652
F4 Gives students chance to reflect on the feedback		0.650			0.881	0.674
Promoting self-directed learning			0.96	0.88		
PS1 Motivates students to study further and deeper in the topic		0.671			0.878	0.787
PS2 Stimulates students to keep up with the literature		0.651			0.875	0.772
PS3 Motivates students to learn independently		0.699			0.855	0.813

^a^ Cronbach’s α >0.70 was taken as an indication of satisfactory reliability of each composite scale

^b^ Item-total correlation values <0.4 indicate that the corresponding item does not correlate well with the composite scale

To assess for the reliability, Cronbach’s alpha was calculated for the total scale and for each composite scale. Cronbach’s alpha was 0.94 and higher for the six scales on the student survey. For the clinical teacher survey, Cronbach’s alpha was 0.88. For the subscales, Cronbach’s alpha was 0.95, 0.94, 0.95, 0.97, 0.96, and 0.96 for teaching and learning environment items, professional attitude toward students items, communication of goals items, evaluation of students items, feedback items, and promoting self-directed learning items, respectively. In both instruments, the item total correlation was above 0.40 for all items within their respective scales. Please refer to Table 2 for explicit details (Table 2).

The inter-scale correlations for the student instruments ranged from 0.72 (*P* < 0.001) between professional attitude toward students and promoting self-directed learning to 0.87 (*P* < 0.001) between teaching and learning environment and evaluation of students (Table 2). For the clinical teacher instrument, the inter-scale correlations ranged from 0.59 (*P* < 0.001) between evaluation of students and feedback to 0.85 (*P* < 0.001) between teaching and learning environment and communication of goals (Table 3).

**Table 3:** Inter-scale correlation† for students’ and clinical tutors evaluation separately

Students’ evaluations of clinical tutors						
	Teaching & Learning environment	Professional attitude towards Students	Communication of Goals	Evaluation of Students	Feedback	Promoting self-directed learning
Teaching & Learning environment	1	0.828^**^	0.852^**^	0.869^**^	0.850^**^	0.818^**^
Professional attitude towards Students		1	0.759^**^	0.789^**^	0.799^**^	0.726^**^
Communication of Goals			1	0.845^**^	0.813^**^	0.816^**^
Evaluation of Students				1	0.877^**^	0.845^**^
Feedback					1	0.862^**^
Promoting self-directed learning						1
Clinical tutor self-evaluation						
Teaching & Learning environment	1	0.826**	0.854**	0.718**	0.791**	0.706**
Professional attitude towards Students		1	0.791**	0.614**	0.719**	0.700**
Communication of Goals			1	0.686**	0.792**	0.739**
Evaluation of Students				1	0.595**	0.731**
Feedback					1	0.690**
Promoting self-directed learning						1

^*^ *P* < 0.05

**P < 0.001

### Number of student evaluations per clinical teacher needed

To have a reliable result about the clinical teacher evaluation, we found that at least four student evaluations were needed for each clinical teacher to reach a reliability of 0.60. On average, we had 11.2 evaluations for each clinical teacher. To achieve a reliability of 0.70 and 0.80, a minimum number of 5 and 6 student assessments per teacher are required, respectively (Tables 4 and 5).

**Table 4:** Number of students’ evaluations needed per clinical tutor for reliable evaluation of clinical tutor teaching qualities

Scales	Cronbach’s α		
	Cronbach’s α of 0.60	Cronbach’s α of 0.70	Cronbach’s α of 0.80
Teaching and learning environment	4	5	6
Professional attitude towards students	4	5	6
Communication of goals	4	5	6
Evaluation of students	4	5	6
Feedback	4	5	6
Promoting self-directed learning	4	5	6

**Table 5:** Estimated reliabilities (Cronbach’s α) at different numbers of students’ evaluation completed per clinical teacherss

Scales	Cronbach’s		
	4 Evaluations	5 Evaluations	6 Evaluations
Teaching and learning environment	0.895	0.856	0.972
Professional attitude towards students	0.853	0.947	0.868
Communication of goals	0.990	0.900	0.963
Evaluation of students	0.956	0.776	0.916
Feedback	0.877	0.771	0.935
Promoting self-directed learning	0.804	0.891	0.978

## Discussion

Medical academic institutes highly emphasize on the expectation from the students towards the end of each clinical rotation. This shifting toward competency based education requires clinical teachers to review and potentially improve their teaching qualities. Our study showed that the modified SETQ instruments can be used for the evaluation of the clinical teachers across medical schools. This study offers pragmatic support for the feasibility and psychometric properties of the modified SETQ instruments for clinical teachers among medical schools. Re-introducing a domain from the SFDP26 to the original SETQ instrument enabled us further to explore the capability of the clinical teachers to stimulate and influence students’ self-directed learning. This domain was not covered by the original SETQ. In addition, adding new questions to the modified SETQ instrument, such as teaching in the ward round, clinics, operating room, and covering all the topics which are in the curriculum, gave us insight what happened with the students in daily practice.

Modified questionnaires such as the ones administered in this study are essential for organizations to achieve the insight of future development of clinical teaching, to improve the quality of clinical instruction and contribute to the medical students’ learning. They give an opportunity for both students and teachers to reflect and improve upon the learning process. Clinical teaching improves when clinical teachers receive feedback from their students. Past research has indicated that this improvement is only as effective as the domains covered by the assessment tool itself; important domains to be included are: teaching, role modeling, providing feedback, being supportive, assigning relevant clinical work, assessing students, and planning teaching activities [16].

This multicenter study found that the modified SETQ instrument is a feasible, reliable, and valid method to evaluate the teaching qualities of clinical teachers. The number of minutes required to complete the questionnaire, and the low number of evaluations needed for reliable assessment indicate the feasibility of the modified SETQ instrument for the evaluation of clinical teachers in different specialties. This finding corresponds with the number of evaluations needed in the original SETQ instrument for anesthesiology and obstetrics and gynecology [7, 8, 17].

The six composite scales raised from the factor analysis of the student evaluation support the construct validity of the instrument. With the clinical teacher self-evaluation (57 records for structuring 25 items), we were not able to conduct a stable factor analysis. However, the validity of both instruments was supported by the item-total correlation and inter-scale correlation which were within predefined limits.

We also found in this study that there was a variation in the quality of teaching among the clinical teachers. Student evaluations revealed differences between the individual clinical teachers in all six domains. The utilization of a modified SETQ system in undergraduate medical education and in a Middle Eastern setting is new approach. To our knowledge, this is the first study that uses the SETQ system for clinical year’s medical students in a Middle Eastern setting.

The SETQ system enables the clinical teachers to evaluate their performance and subsequently could lead to improve the quality of teaching among the clinical teachers with poor performance [18]. This study focuses on the psychometric properties of the modified SETQ system, however, future research should focus on the effectiveness of the SETQ system in improving the quality of teaching.

### Limitations of the study

While questionnaire-based studies on a large sample size such as this one are often an effective means of extrapolating data, they also lend themselves to some potential limitations. Firstly, the 42% student response rate may be considered medium to low. However, this can be overcome in future studies by introducing the paper-based method to all students. The majority of the low rates came from Intermediate Cycle-3 (IC3) denoted in Table 6, where we introduced the online system while students were doing their respective rotations in hospitals. A possible reason that the paper-based method was more effective is that students completed it in person and were less likely to forget. It is also likely that seeing other students participate encouraged individuals to also engage in the study. On the other hand, the online method relies on students independently filling out the evaluation forms and remembering to log online in their own time.

**Table 6:** Rotations in departments across hospitals

	Hospital A	Hospital B	Hospital C	Hospital D
Rotations in Departments	Medicine	Surgery	Surgery	Psychiatry
	Surgery	Medicine	Medicine	
	Psychiatry	Obstetrics & Gynaecology	Paediatrics	
	Paediatrics		Obstetrics & Gynaecology	
	Pathology			
	Obstetrics & Gynaecology			
Sub-specialties in each Department	Neurology, Cardiology, Urology, and Endocrinology			

A second significant limitation was that these questionnaires were entirely student-centered. While this is an important aspect of examining learning, future studies might benefit from using 360° multisource feedback that involves both clinical teachers and their colleagues as well. Another limitation is that although the surveys were anonymous, full anonymity may not be possible to achieve due to the small department size in some locations, where it was difficult to fully report findings without compromising anonymity. This is an important issue as studies have found that anonymous ratings tend to be lower than their transparent counterparts [14].

Finally, another limitation is that the modified SETQ has not been used among students in different medical schools and in different settings. Replicating similar work with other medical schools and in different settings may be advisable.

## Conclusion

Our modified SETQ questionnaire was found to be both reliable and valid, and was implemented successfully across various departments and specialties in different hospitals in the Kingdom of Bahrain. This modified SETQ tool was found to be applicable in our settings and will be used in the future to evaluate clinical teachers in Bahrain. Future research should focus on the effectiveness of SETQ to contribute to improvement of teaching.

## Additional files

Additional file 1: Study Questionnaire. (DOCX 1773 kb)
Additional file 2: Chart 1. (DOCX 79 kb)
Additional file 3: Master sheet data. (XLS 596 kb)

## Abbreviations

IC3: Intermediate Cycle-3
KMO: Kaiser-Meyer-Olkin
RCSI-Bahrain: Royal College of Surgeons in Ireland- Bahrain
SETQ: System for Evaluation of Teaching Qualities
SFDP26: Stanford Faculty Development Program
