# Supplementary material for: Analysis of psychometric properties of the modified SETQ tool in undergraduate medical education
Source: BMC Med Educ. 2017 Mar 16;17:56. doi: 10.1186/s12909-017-0893-4 (PMC5356325; doi:10.1186/s12909-017-0893-4)
Supplement: Additional file 1: — Study Questionnaire. (DOCX 1773 kb) [file 12909_2017_893_MOESM1_ESM.docx]

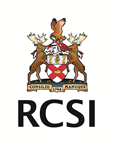
**Evaluation of Clinical Tutor by Students**

**
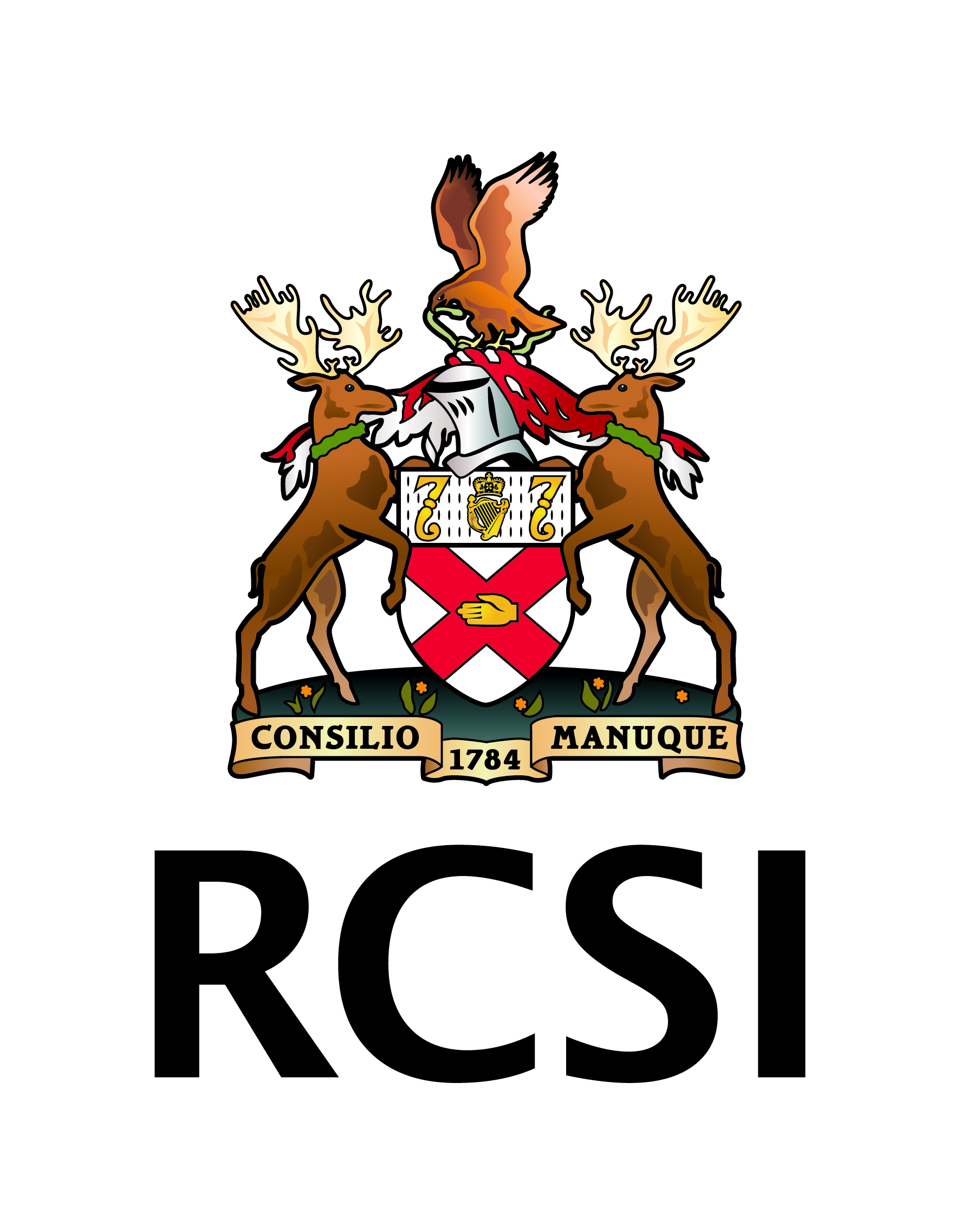
**

Student Name: Date of Rotation:

Student ID: Cycle Year:

Clinical Tutor Name: Hospital Name:

**Please Tick (🗸) the applicable box using the below key:**

1. Strongly disagree **2-** Disagree **3-** Neutral

**4-** Agree **5-** Strongly agree **6-** Unable to assess

| **Sr.**  **No** | **Teaching & Learning environment** | **1**  Strongly disagree | **2**  Disagree | **3**  Neutral | **4**  Agree | **5**  Strongly agree | **6**  Unable to assess |
| --- | --- | --- | --- | --- | --- | --- | --- |
| **Q1.** | Encourages students to participate actively in discussions |  |  |  |  |  |  |
| **Q2.** | Stimulates students to bring up problems |  |  |  |  |  |  |
| **Q3.** | Keeps to teaching goals; avoids digressions |  |  |  |  |  |  |
| **Q4.** | Prepares well for teaching presentations and talks |  |  |  |  |  |  |
| **Q5.** | Teaches on ward rounds, at clinics, and operating room |  |  |  |  |  |  |
| **Q6.** | Covering all the topics which are in the curriculum |  |  |  |  |  |  |
| **Professional attitude towards Students** | | | | | | |  |
| **Q7.** | Listens attentively to students |  |  |  |  |  |  |
| **Q8.** | Is respectful towards students |  |  |  |  |  |  |
| **Q9.** | Is available regularly for the students |  |  |  |  |  |  |
| **Q10.** | Is easily approachable for discussions |  |  |  |  |  |  |
| **Communication of Goals** | | | | | | |  |
| **Q11.** | States learning goals clearly |  |  |  |  |  |  |
| **Q12.** | Prioritizes learning goals and topics |  |  |  |  |  |  |
| **Q13.** | Debriefing the learning goals periodically |  |  |  |  |  |  |
| **Evaluation of Students** | | | | | | |  |
| **Q14.** | Evaluates student’s specialty knowledge regularly |  |  |  |  |  |  |
| **Q15.** | Evaluates student’s analytical abilities regularly |  |  |  |  |  |  |
| **Q16.** | Evaluates student’s application of knowledge to specific patients |  |  |  |  |  |  |
| **Q17.** | Evaluates student’s medical skills regularly |  |  |  |  |  |  |
| **Q18.** | Evaluates student’s, communication, and professionalism during patient encounter |  |  |  |  |  |  |
| **Feedback** | | | | | | |  |
| **Q19.** | Regularly gives constructive feedbacks to students |  |  |  |  |  |  |
| **Q20.** | Explains why students are incorrect |  |  |  |  |  |  |
| **Q21.** | Offers suggestions for improvement |  |  |  |  |  |  |
| **Q22.** | Gives students chance to reflect on the feedback |  |  |  |  |  |  |
| **Promoting self-directed learning** | | | | | | |  |
| **Q23.** | Motivates students to study further and deeper in the topic |  |  |  |  |  |  |
| **Q24.** | Stimulates students to keep up with the literature |  |  |  |  |  |  |
| **Q25.** | Motivates students to learn independently |  |  |  |  |  |  |
