# Supplementary material for: Analysis of psychometric properties of the modified SETQ tool in undergraduate medical education
Source: BMC Med Educ. 2017 Mar 16;17:56. doi: 10.1186/s12909-017-0893-4 (PMC5356325; doi:10.1186/s12909-017-0893-4)
Supplement: Additional file 2: — Chart 1. (DOCX 79 kb) [file 12909_2017_893_MOESM2_ESM.docx]

**Chart 1: Student and Clinical Teacher Characteristics**
